# Cross-Virus RNA Language Models for Within-Family RNA–RNA Interaction Prediction in SARS-CoV-2 and Porcine Deltacoronavirus

**DOI:** 10.3390/ijms27177565

**Published:** 2026-08-24

**Authors:** Xu Yang, Weiwei Shen, Yixue Li, Liucun Zhu, Tao Huang

**Affiliations:** 1 Shanghai Institute of Nutrition and Health, University of Chinese Academy of Sciences, Chinese Academy of Sciences, Shanghai 200031, China; yangxu2025@sinh.ac.cn (X.Y.); sww1025@shu.edu.cn (W.S.); li_yixue@gzlab.ac.cn (Y.L.); 2 School of Life Sciences, Shanghai University, Shanghai 200444, China; 3 Guangzhou National Laboratory, Guangzhou 510000, China; 4 The Guangdong-Hong Kong-Macau Joint Laboratory for Cell Fate Regulation and Diseases, Guangzhou Laboratory, GMU-GIBH Joint School of Life Sciences, Guangzhou Medical University, Guangzhou 510000, China; 5 Key Laboratory of Systems Health Science of Zhejiang Province, School of Life Science, Hangzhou Institute for Advanced Study, University of Chinese Academy of Sciences, Hangzhou 310024, China

**Keywords:** RNA large language models (RNA LLMs), cross-virus prediction, porcine delta coronavirus, SARS-CoV-2, RNA–RNA interaction (RRI)

## Abstract

RNA large language models (RNA LLMs) show potential for predicting viral RNA–RNA interactions (RRIs), but their ability to transfer between viruses remains unclear. Here, we evaluated three nucleotide language models using vRIC-seq-derived RRI datasets from two members of the Coronaviridae family, SARS-CoV-2 and porcine deltacoronavirus (PDCoV). We compared within-virus five-fold cross-validation with bidirectional cross-virus testing. DNABERT achieved within-virus AUC values of 0.9595 for PDCoV and 0.9741 for SARS-CoV-2. Under cross-virus transfer, its AUC decreased to 0.8372 when trained on PDCoV and tested on SARS-CoV-2 and to 0.8116 in the reverse direction, with corresponding F1 scores of 0.7847 and 0.7617. RNAErnie retained similar but slightly lower cross-virus discrimination, whereas the Nucleotide Transformer approached random discrimination. These results provide a two-virus proof of concept that sequence-based models can retain partially shared discriminative signals across coronavirus genera. They do not establish functional, tropism-related, or receptor-dependent transferability, and broader evaluation across additional viruses and orthogonal structural validation will be required before application to emerging-virus screening.

## 1. Introduction

Porcine deltacoronavirus (PDCoV) is a globally distributed enteric pathogen causing severe diarrhea and mortality in neonatal piglets, inflicting substantial economic losses [1]. Beyond swine, PDCoV infects poultry [2] and has been detected in pediatric patients with acute febrile illness, confirming zoonotic spillover [3]. Genomically a δ-coronavirus, PDCoV has the smallest genome among known coronaviruses [4].

PDCoV and SARS-CoV-2 belong to different genera (δ versus β) within the Coronaviridae family. SARS-CoV-2 uses ACE2 as an established entry receptor, and its spike is proteolytically primed by host proteases that include furin and TMPRSS2 in human airway cells [5,6,7]. PDCoV entry is not adequately described by a single receptor–protease pair. APN can facilitate entry but is not universally required, whereas heparan sulfate and sialic acid act as attachment factors [8,9,10,11]. Trypsin can enhance attachment, spike-mediated fusion, and productive infection in particular cell systems, but PDCoV can also use an endosomal cathepsin-dependent pathway [12,13]. These biological differences motivate a carefully bounded cross-genus comparison. They do not establish that the two viruses share functional RNA–RNA interaction (RRI) elements or entry-related mechanisms. Both genomes nevertheless use long-range RNA contacts in virion packaging and coronavirus RNA regulation [14,15].

Computational RRI prediction has progressed from feature-based classifiers to deep neural networks and Transformer-based nucleotide language models. Classical methods depend on manually designed sequence representations, whereas deep models learn task-specific features directly from the sequence. Transformer models provide contextual representations through self-attention and large-scale pre-training, making them plausible candidates for modeling non-local sequence associations. However, self-attention does not by itself demonstrate capture of tertiary RNA interactions. Covariance models used for RNA-family annotation detect conservation and covariation within homologous sequence families, whereas the present task classifies whether two fragments form a contact pair derived from virion RNA in situ conformation sequencing (vRIC-seq). These tasks are related but not equivalent, and this study did not perform a head-to-head benchmark against Rfam.

We therefore compared traditional machine-learning baselines, CNN/RNN-based models, and three pre-trained nucleotide language models under a unified within-virus and bidirectional cross-virus evaluation framework. Detailed encoding procedures and model architectures are provided in the Section 4.

Our analysis uses genomic sequences and labels derived from contacts in mature virions. It evaluates two-virus, within-family transfer of a paired-fragment classifier and does not test host tropism, receptor-dependent entry, zoonotic spillover, transmissibility, or pandemic risk.

## 2. Results

### 2.1. Traditional Machine Learning Baselines Exhibit Classifier- and Encoding-Dependent Performance

As shown in Table 1, under one-hot encoding, model performance was primarily influenced by classifier type. With one-hot encoding, random forest produced the highest AUC (0.8543), showing that the ensemble tree model retained useful signal even from sparse nucleotide vectors. In contrast, KNN achieved the lowest AUC (0.8070) under one-hot encoding, which is consistent with the difficulty of using distance-based voting in a high-dimensional sparse representation space.

**Figure 1:**
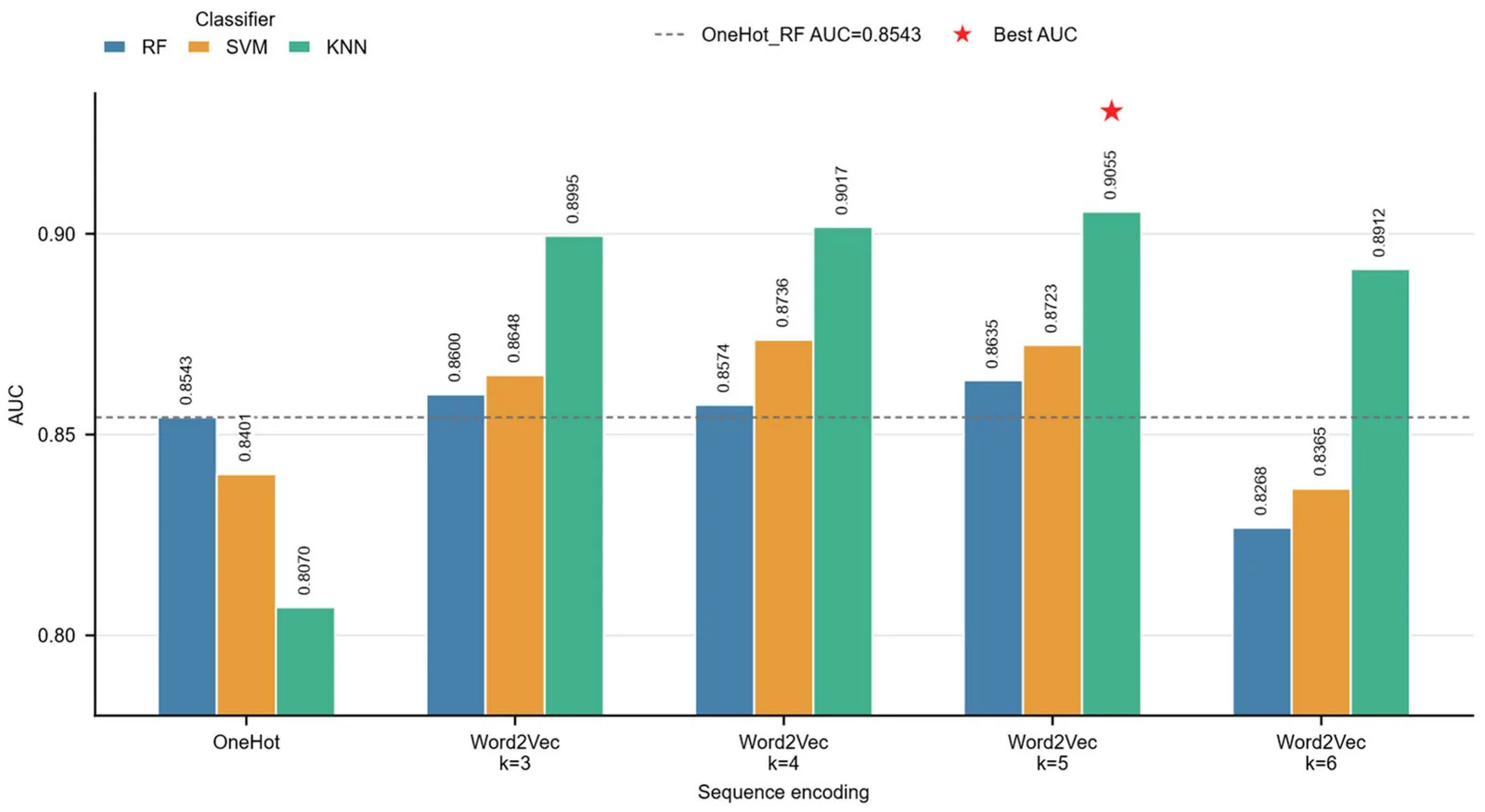
Performance comparison of traditional machine learning models under OneHot and Word2Vec k-mer encodings. The figure shows AUC values obtained by RF, SVM, and KNN classifiers on the PDCoV dataset using five-fold cross-validation. The dashed line indicates the best baseline model under OneHot encoding (OneHot + RF; AUC = 0.8543). Numeric labels indicate the AUC values for all bars. The red star marks the best traditional machine-learning configuration, Word2Vec k = 5 with KNN (AUC = 0.9055).

Using Word2Vec k-mer representations improved the traditional machine learning baselines, and the clearest gain was observed for KNN. Across Word2Vec representations with k = 3 to k = 6, KNN achieved the highest AUC within each k-mer setting, reaching the overall best AUC among traditional machine learning methods at k = 5 (AUC = 0.9055; Figure 1 and Table 1). KNN also showed higher recall with Word2Vec features, suggesting that the continuous k-mer space made neighboring samples more informative for identifying interaction-positive pairs.

This tendency reduces false positives but also misses some true positive samples. SVM showed generally moderate performance across all settings. Thus, performance depended on the interaction between representation choice, distance or split criterion, and the classifier decision boundary.

### 2.2. deepRAM Further Improves Prediction Performance over Traditional Machine Learning Baselines

**Figure 2:**
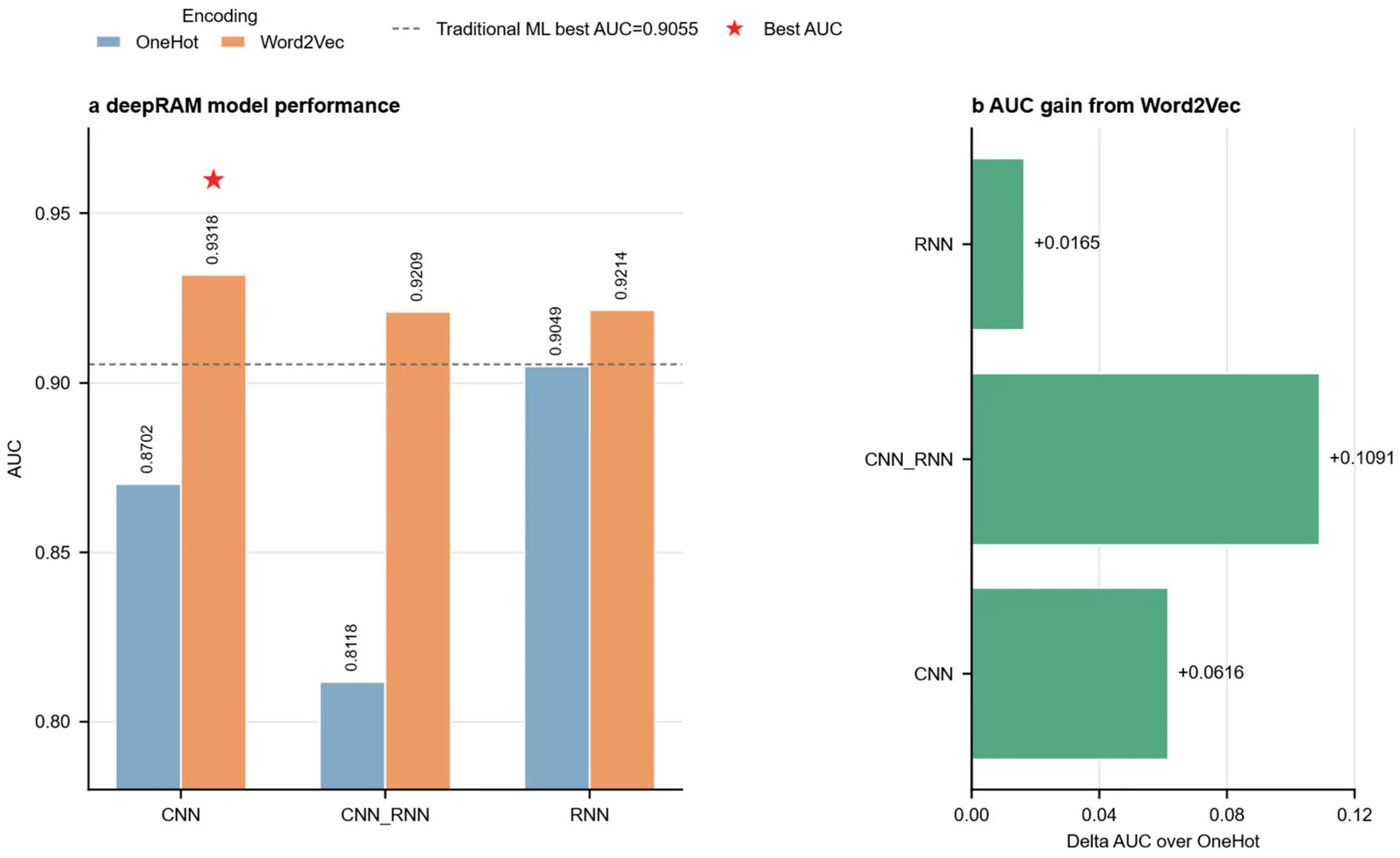
Performance comparison of deepRAM architectures under different encoding methods. (**a**) AUC of CNN, CNN_RNN, and RNN deepRAM architectures under one-hot and Word2Vec representations. The dashed line indicates the AUC of the best traditional machine learning model (0.9055). Numeric labels show the AUC values for all bars, and the red star marks the best deepRAM configuration, Word2Vec with CNN (AUC = 0.9318). (**b**) AUC improvement of Word2Vec over one-hot encoding across deepRAM architectures. All results are 5-fold cross-validation averages on the PDCoV dataset.

Beyond traditional machine learning baselines, we further evaluated the combined effects of different sequence encoding methods and deep learning architectures within the deepRAM framework. deepRAM’s performance was influenced not only by network architecture but also substantially by the input representation method. Compared with traditional classifiers, the primary advantage of deepRAM lies in its ability to automatically learn local or sequential features from input sequence representations rather than relying only on manually specified decision boundaries. Among all deepRAM combinations, the CNN architecture with Word2Vec representation achieved the highest AUC (0.9318; Figure 2a and Table 2), surpassing the best traditional machine learning model (KNN with k = 5 Word2Vec, AUC = 0.9055). This result suggests that, after k-mer representations organize the input space, convolutional filters can add useful local-pattern information for RRI prediction. The effect of encoding method on deepRAM performance was more pronounced than simply changing the architecture. Word2Vec representation improved AUC across all three architectures (CNN, CNN_RNN, and RNN), with the largest gain observed for CNN_RNN (+0.1091) and a substantial increase for CNN (+0.0616; Figure 2b). These results indicate that dense k-mer vectors provided a more useful input than sparse one-hot matrices for the deepRAM models in this task.

The architecture comparison also showed that greater model complexity did not automatically improve performance. Under one-hot encoding, RNN was the best-performing deepRAM architecture (AUC = 0.9049), approaching the optimal traditional machine learning baseline (Table 2). This likely reflects the recurrent structure’s ability to integrate contextual information along the sequence direction, partially compensating for the lack of continuous semantic relationships in one-hot representations. Conversely, under Word2Vec representation, CNN outperformed both RNN and CNN_RNN, suggesting that when the input already contains distributed k-mer-level information, local pattern extraction may be more effective than further modeling long-range sequential dependencies. CNN_RNN did not outperform the best single architecture under either encoding method and was weakest with one-hot input, suggesting that the hybrid model added training complexity without a consistent gain for this dataset.

The CNN architecture with Word2Vec representation constituted the optimal deepRAM configuration. Its advantage was reflected in high AUC, F1-score, and accuracy together, with no large trade-off between precision and recall. This result suggests that, for this RRI prediction task, the benefit of deep learning models primarily arises from the appropriate match between sequence representation and local feature extraction architecture, rather than from simply increasing the number of network modules.

### 2.3. Pre-Trained Language Models Achieve the Strongest Within-Dataset Performance

**Figure 3:**
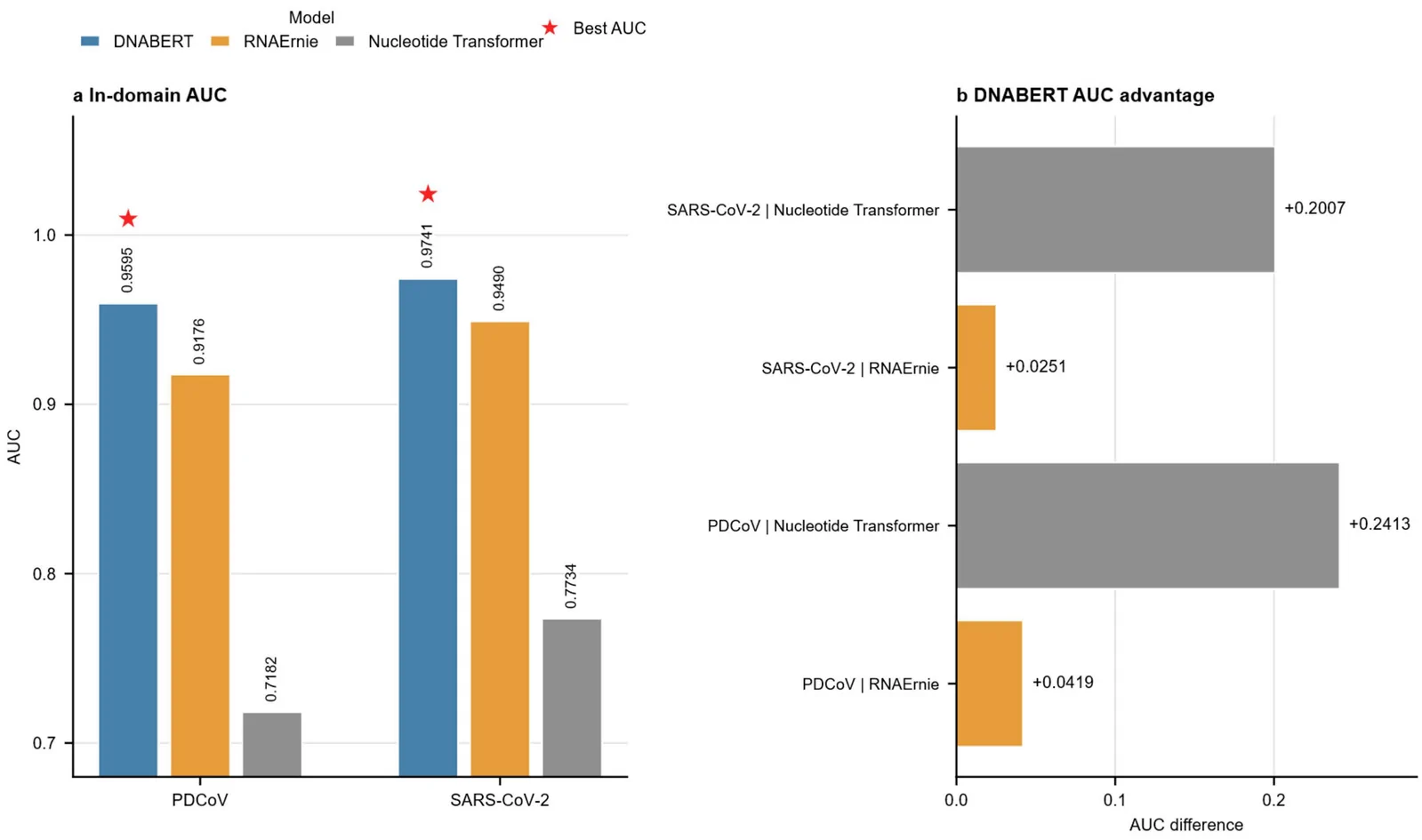
Within-dataset prediction performance of pre-trained language models on PDCoV and SARS-CoV-2 datasets. (**a**) Five-fold cross-validation AUC values for DNABERT, RNAErnie, and NT on the PDCoV and SARS-CoV-2 datasets. Numeric labels indicate the AUC values for all bars. Red stars mark the best model within each dataset; DNABERT achieved the highest AUC on both PDCoV (0.9595) and SARS-CoV-2 (0.9741). (**b**) AUC advantage of DNABERT over RNAErnie and NT. All metrics are within-dataset 5-fold cross-validation averages.

Following traditional machine learning and deepRAM models, we compared the within-dataset five-fold cross-validation performance of three pre-trained language models on both the PDCoV and SARS-CoV-2 datasets. The ranking of model performance was consistent across both datasets: DNABERT performed best, followed by RNAErnie, with the NT ranking lowest (Figure 3a and Table 3). DNABERT achieved AUC values of 0.9595 and 0.9741 on the PDCoV and SARS-CoV-2 datasets, respectively, outperforming all non-pre-trained models from the traditional machine learning and deepRAM evaluations.

DNABERT’s advantage stems from the alignment between its input granularity, encoding structure, and the present task. Rather than modeling individual bases, DNABERT segments sequences into k-mer tokens and uses the [CLS] representation at the sequence start to aggregate global classification information. Multiple Transformer blocks jointly establish contextual relationships among different k-mer fragments through multi-head self-attention. For RRI prediction, classification labels often depend on short fragment combinations, local complementarity, or co-occurring motif-like signals across two sequences, rather than the isolated state of individual bases. Thus, k-mer tokens treat local fragments as basic semantic units, while self-attention globally weights the relationships among these fragments, a structural approach better suited for integrating local patterns and contextual evidence than CNN’s fixed windows or RNN’s sequential compression. DNABERT not only achieved the highest AUC but also maintained balanced precision, recall, and F1-score, indicating that its improvement did not come at the cost of sacrificing one class for another (Table 3).

RNAErnie ranked second on both datasets, confirming that RNA-specific pre-training provides effective sequence representations. Compared with DNABERT, RNAErnie’s design focus extends beyond standard masked language modeling to incorporate three-level masking (base, subsequence, and motif) and RNA type information as auxiliary tokens during both pre-training and type-guided fine-tuning. This design facilitates the learning of RNA motifs, structural/functional categories, and representation differences across RNA types, enabling clear improvements over non-pre-trained models. The performance gap between RNAErnie and DNABERT was smaller on the SARS-CoV-2 dataset, where RNAErnie was 0.0251 lower than DNABERT, compared with the PDCoV dataset, where it was 0.0419 lower, as shown in Figure 3b. However, in the present task, samples are primarily virus genome fragments whose labels are determined by whether two sequences interact, rather than directly by RNA type classification or low-level RNA ontology. The advantage of RNAErnie’s type-guided design may therefore not have been fully amplified. By contrast, DNABERT’s more direct k-mer fragment representation and [CLS]-level binary classification fine-tuning pathway align more closely with the current interaction discrimination task.

NT showed notably weaker performance in this task, with AUC deficits of 0.2413 and 0.2007 relative to DNABERT on PDCoV and SARS-CoV-2, respectively (Figure 3b). NT’s original design targets longer genomic contexts, learning general nucleotide representations from large-scale human or multi-species genome sequences, with demonstrated transferability in promoter, splice site, enhancer, and chromatin-related tasks. This long-context, general genomic feature learning is valuable for metagenomic region identification but may be less suited to the shorter, paired viral RRI fragments in this study. The representations learned by NT may be biased toward genomic functional elements, long-range regulatory regions, or cross-species conserved signals, whereas the classification boundary in this task depends more on the combinatorial relationship of local interaction fragments between two RNA sequences. NT’s recall exceeding precision on SARS-CoV-2 also suggests that while it can identify some potential positive samples, its positive classification is more permissive, resulting in less balanced overall classification.

All three pre-trained models achieved higher AUC on the SARS-CoV-2 dataset than on the PDCoV dataset, but their relative ranking remained unchanged. This indicates that the performance differences are not incidental to a single dataset but are reproducible across both viral datasets. Collectively, the within-dataset results establish DNABERT as the strongest pre-trained language model in this study. Its advantage does not stem from having the largest parameter count, but from the architectural pathway formed by k-mer tokenization, contiguous fragment masking, self-attention-based context aggregation, and [CLS] classification representation, which together constitute a structure better suited for interaction discrimination.

### 2.4. DNABERT Retains the Best Generalization Ability in Cross-Dataset Evaluation

**Figure 4:**
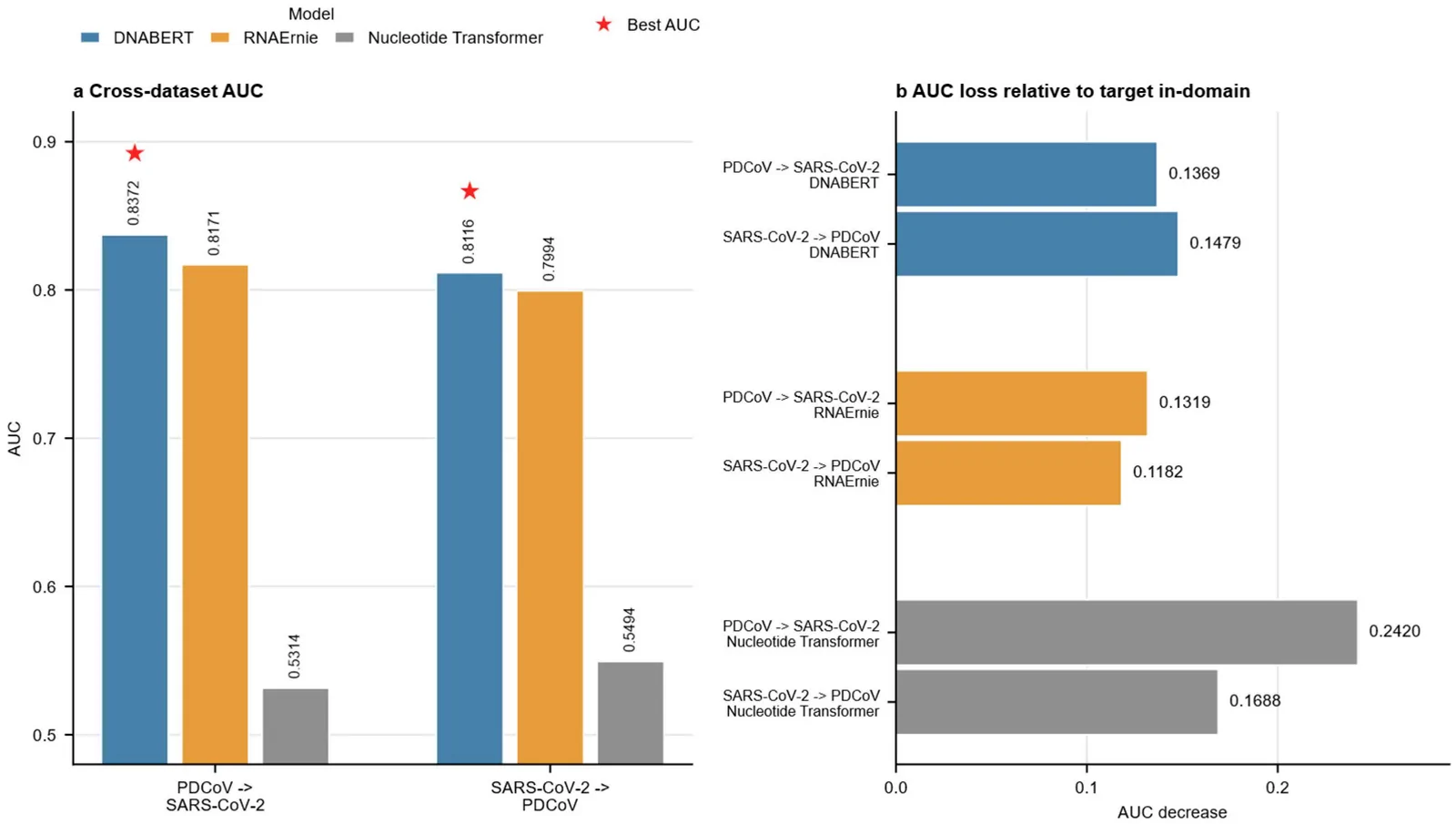
Cross-dataset generalization performance of pre-trained language models. (**a**) Cross-virus transfer AUC values for DNABERT, RNAErnie, and Nucleotide Transformer when training on PDCoV and testing on SARS-CoV-2, or training on SARS-CoV-2 and testing on PDCoV. Numeric labels indicate the AUC values for all bars. Red stars mark the best model in each transfer direction. (**b**) Decrease in cross-dataset AUC relative to the target dataset’s within-dataset AUC for each model. All cross-dataset results were obtained by training on one dataset and testing on the other; target dataset within-dataset AUC values are the 5-fold cross-validation results of the corresponding model on that target dataset.

Cross-dataset evaluation is stricter than within-dataset cross-validation because it tests whether a model retains RRI signal across coronaviruses instead of only matching the composition, sampling pattern, or local context of one dataset. PDCoV and SARS-CoV-2 are both positive-sense RNA viruses, but they differ in genome sequence, local structural context, and experimental origin. If a model trained on one virus remains discriminative on the other, the learned features are unlikely to be only virus-specific memorization and may include transferable signals such as local complementarity, conserved contacts, or motif-like fragment combinations. Under this more stringent setting, all models exhibited lower performance than their respective within-dataset five-fold cross-validation results, consistent with a distribution shift between the two virus datasets. Nevertheless, DNABERT maintained the highest absolute AUC in both transfer directions: 0.8372 for PDCoV to SARS-CoV-2 and 0.8116 for SARS-CoV-2 to PDCoV (Figure 4a).

DNABERT’s cross-dataset advantage suggests that transfer in this task depends less on model scale alone than on the match between token representation, attention-based modeling, and RRI patterns. DNABERT uses k-mers as basic tokens, aggregates sequence-level classification information through the [CLS] representation, and enables non-local associations among different k-mer fragments via multi-head self-attention. For RRI prediction, the relevant signal often lies in short sequence fragments, local complementarity, and combinations of fragments rather than in isolated bases. Although PDCoV and SARS-CoV-2 differ in specific sequences, some fragment combination patterns related to RNA pairing, folding constraints, or conserved functional regions may recur across coronavirus RNAs. DNABERT maintained AUC exceeding 0.8 in both transfer directions, retaining 85.95% and 84.59% of the target dataset’s within-dataset AUC, respectively (Table 4), indicating that its learned representations are not entirely dependent on the training virus.

RNAErnie achieved cross-virus AUC values of 0.8171 and 0.7994, close to but slightly below the corresponding DNABERT results (Table 4). This performance is consistent with, but does not by itself prove, a contribution of RNA-oriented pre-training to transfer. Motif-aware pre-training may retain useful RNA sequence context under distribution shift, whereas the paired-fragment classification boundary may also depend on tokenization and model–task alignment.

In contrast, NT achieved AUC values of only 0.5314 and 0.5494 in the two transfer directions, approaching random discrimination (Table 4). This result does not imply that large-scale nucleotide pre-training lacks value, but rather that the strengths of general genomic foundation models must align with the specific task type. NT’s design emphasizes kilobase-scale genomic contexts, cross-population or multi-species genome representations, and functional element identification in promoter, splice site, enhancer, and chromatin-related tasks. It excels at functional annotation and regulatory signal modeling within long genomic regions, whereas the cross-virus RRI prediction in this study depends more heavily on pairwise relationships between shorter fragments. NT may have learned broad nucleotide-level and genomic-element representations that do not reliably capture the local complementarity, short motif combinations, and binary pairing relationships required for coronavirus RNA internal interaction prediction. Thus, NT’s low cross-domain AUC supports a core finding of this study: cross-virus generalization is not determined by pre-training data volume or model scale alone, but by the alignment among pre-training objective, token granularity, and the biological task.

The retained cross-virus discrimination is consistent with partially shared sequence-level signals, which may include local complementarity, family-conserved motifs, virus-specific patterns, or dataset-related regularities that cannot be separated by the present design. The approximately 0.12–0.15 reduction in AUC relative to within-virus validation indicates partial rather than complete transfer. These results support candidate-contact prioritization as a hypothesis-generation use, but they do not establish functional equivalence of individual RRI elements or reliability in an uncharacterized virus.

### 2.5. UMAP Visualization Reveals Class- and Dataset-Associated Organization of DNABERT Representations

**Figure 5:**
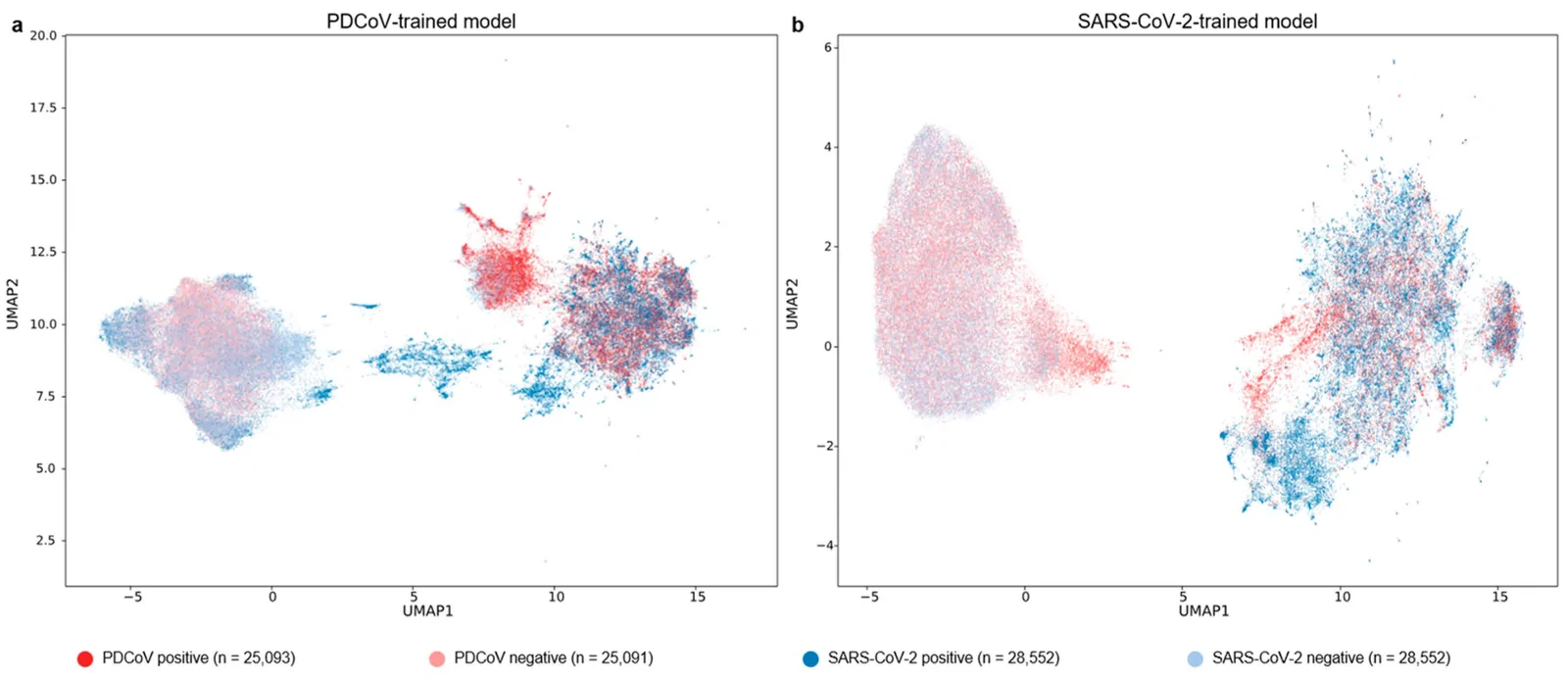
UMAP visualization of DNABERT representations learned from PDCoV and SARS-CoV-2 RNA–RNA interaction datasets. (**a**) UMAP projection of five-fold-averaged final-layer [CLS] embeddings generated by DNABERT models trained on the PDCoV dataset. (**b**) Corresponding projection generated by DNABERT models trained on the SARS-CoV-2 dataset. Each point represents one paired RNA-fragment sample. Dark red and light red denote RRI-positive (*n* = 25,093) and RRI-negative (*n* = 25,091) PDCoV samples, respectively; dark blue and light blue denote RRI-positive and RRI-negative SARS-CoV-2 samples, respectively (*n* = 28,552 for each class). UMAP was applied to the 768-dimensional embeddings using 15 nearest neighbors, a minimum distance of 0.1, two output dimensions, and a random seed of 42. The two projections were fitted independently; therefore, their coordinate scales and inter-cluster distances are not directly comparable.

To complement the scalar classification metrics, we examined how DNABERT organized PDCoV and SARS-CoV-2 samples in its learned representation space. For each training dataset, final-layer [CLS] embeddings generated by the five cross-validation models were averaged and projected using Uniform Manifold Approximation and Projection (UMAP) (Figure 5). In both projections, negative samples from PDCoV and SARS-CoV-2 were concentrated in a broad overlapping region, whereas positive samples were enriched across several structures with substantial inter-virus mixing. The overall topology differed between the PDCoV-trained and SARS-CoV-2-trained models, indicating that the source training dataset influenced the organization of the learned representations. These projections provide qualitative evidence that the embeddings contain class- and dataset-associated structure while retaining partially shared sequence-level patterns across the two viral datasets. Because the two UMAP models were fitted independently and UMAP does not preserve global distances, absolute coordinates and inter-cluster distances were not compared across panels.

## 3. Discussion

We compared traditional machine-learning, deep-learning, and Transformer-based nucleotide language models for within-virus RRI classification and bidirectional transfer between SARS-CoV-2 and PDCoV. DNABERT produced the strongest overall discrimination, but the cross-virus results are most appropriately interpreted as a two-virus, within-family proof of concept.

The performance differences among model classes should not be interpreted as a hierarchy of biological realism. DNABERT’s advantage may reflect task-to-model alignment: overlapping k-mer tokenization and paired-sequence contextualization may be particularly compatible with fragment-level binary classification. Self-attention can model non-local statistical dependencies, but it does not directly identify tertiary contacts without orthogonal structural evidence.

Cross-virus testing produced lower performance than within-virus validation. The reduction from AUC values of 0.9595–0.9741 to 0.8116–0.8372 indicates incomplete transfer under dataset shift. The small directional difference should be treated as dataset-specific until reproduced across additional viruses and controlled for sequence composition, interaction-site distribution, and sampling depth. RNAErnie’s similar but lower discrimination and the near-random Nucleotide Transformer results further show that model scale or broad genomic pre-training alone does not ensure transfer.

The UMAP analysis provided a complementary qualitative view of the DNABERT representation space. Class-associated organization and partial inter-virus mixing are consistent with embeddings that contain both dataset-associated structure and partially shared sequence-level patterns. However, the projections do not identify whether these signals arise from general base-pairing chemistry, virion-packaging constraints, family-conserved motifs, or dataset bias. Because the two UMAP models were fitted independently and global distances are not preserved, coordinates and inter-cluster distances cannot be compared across panels. UMAP therefore does not establish structural or functional conservation and cannot compensate for the two-virus study design.

The sequence-only model does not directly represent receptor use, protease activation, tissue context, or host factors. SARS-CoV-2 and PDCoV differ substantially in entry biology [5,6,10,12], and indirect associations arising from virus-specific sequence evolution cannot be excluded. Moreover, vRIC-seq labels describe contacts in mature virions. The predictions should therefore be interpreted as sequence signals associated with processed virion RNA-contact maps, not as evidence of entry-related, tropism-level, or functionally necessary mechanisms.

The published contact matrices were processed using the same interaction-score rule for both viruses, but we did not perform additional RPM normalization, abundance-based downsampling, coverage-matched negative sampling, or an abundance-specific sensitivity analysis. Residual associations with fragment abundance or local coverage may therefore remain. Accordingly, the models predict labels derived from processed vRIC-seq contact maps rather than expression-independent structural determinants. Future analyses should include abundance-only baselines, coverage-matched controls, and sensitivity tests across normalization schemes.

A two-virus comparison cannot establish a general rule even across the Coronaviridae family or determine whether transfer is driven by spike, non-structural, or other genomic regions. Additional within-genus and cross-genus controls, region-stratified evaluation, and leave-one-virus-out testing across a multi-virus panel are required. Domain-adversarial training, parameter-efficient adapters, and few-shot calibration could then be evaluated as methods for reducing virus-of-origin information while preserving interaction-label signal. These comparisons and methods were not performed in the present study.

A staged validation strategy is also needed. Concordantly predicted, high-confidence contacts could first be prioritized and compared with available SHAPE-based accessibility profiles or experimentally resolved RNA structures where coverage overlaps. Selected contacts could then be tested by targeted proximity-ligation or compensatory-mutagenesis approaches in an infected-cell or replicon system, assessing both physical contact and effects on replication or viral fitness. This roadmap was not followed here. The present model is not a predictor of spillover, transmissibility, or pandemic risk. Its defensible use is limited to prioritizing candidate intraviral contacts for subsequent structural and functional testing within a broader virological workflow.

## 4. Materials and Methods

**Figure 6:**
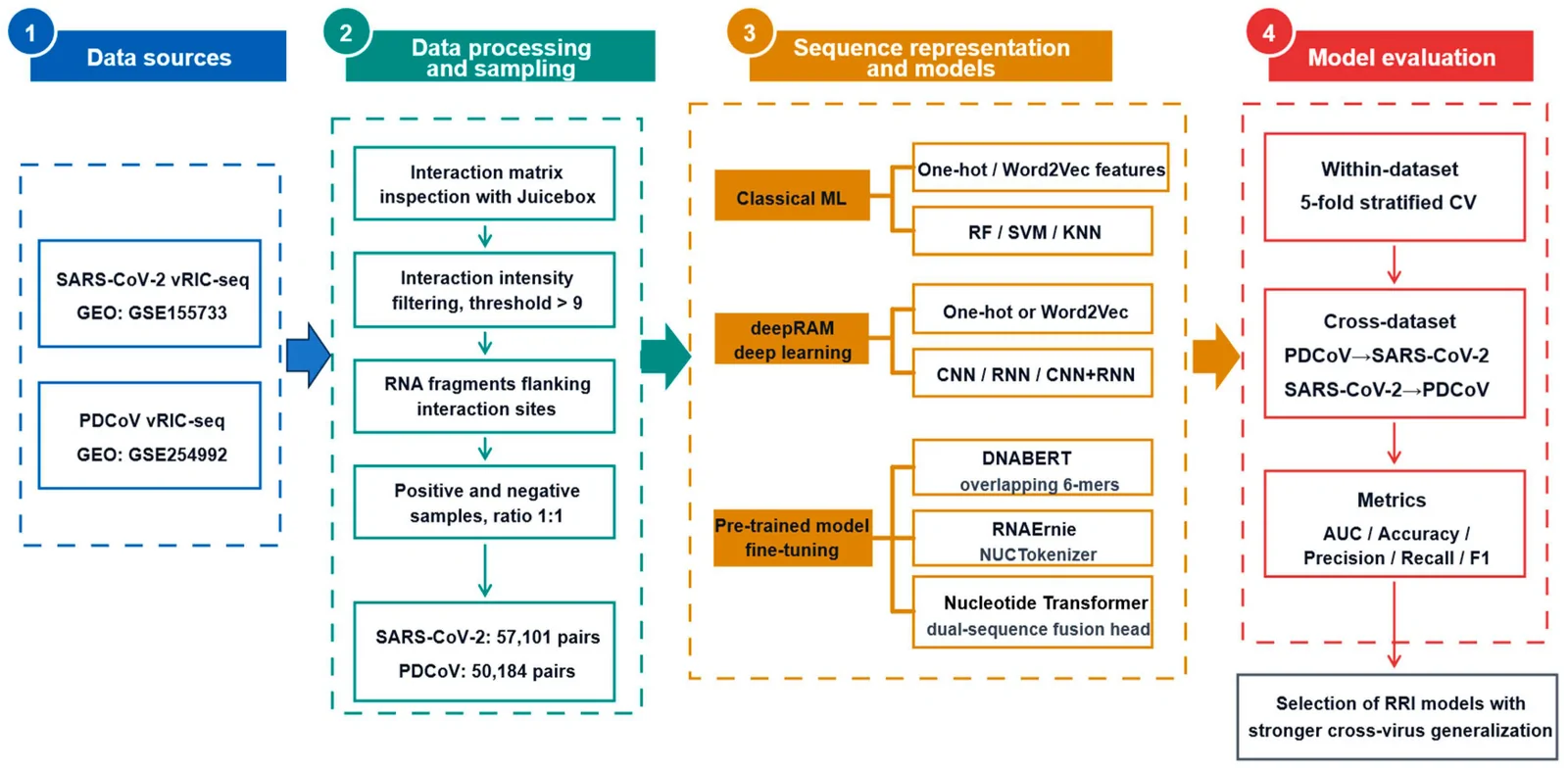
Overall technical pipeline for coronavirus RRI prediction. Public vRIC-seq datasets for SARS-CoV-2 (GSE155733) and PDCoV (GSE254992) were used to construct the RRI prediction task. The interaction matrix was inspected using Juicebox, and interaction pairs with a contact strength greater than 9 were retained as high-confidence sites. RNA fragments flanking the interaction sites were extracted to construct balanced datasets with a 1:1 positive-to-negative ratio. For sequence representation and model construction, one-hot encoding, Word2Vec k-mer embeddings, model-specific tokenization strategies, and pre-trained nucleic acid language model representations were evaluated. Model performance was assessed through five-fold stratified cross-validation within each dataset and bidirectional cross-dataset generalization experiments between PDCoV and SARS-CoV-2, using AUC, accuracy, precision, recall, and F1-score as evaluation metrics.

We constructed a complete technical pipeline for coronavirus RRI prediction, encompassing vRIC-seq data acquisition, sample generation, sequence representation, model fine-tuning, and performance evaluation (Figure 6). RNA interaction sites were extracted from vRIC-seq datasets for SARS-CoV-2 and PDCoV, and interaction pairs with high confidence were retained based on interaction strength thresholds. RNA sequence fragments flanking the interaction sites were extracted to construct balanced binary classification datasets with equal proportions of positive and negative samples. At the model construction stage, we compared the RRI prediction performance of traditional machine learning models, the deepRAM deep learning architecture, and pre-trained nucleic acid language models. Using within-dataset five-fold stratified cross-validation and bidirectional cross-dataset generalization experiments, we systematically evaluated each model’s ability to recognize coronavirus RRI patterns and its cross-virus generalization capability.

### 4.1. Data Sources

We used two publicly available coronavirus genomic RRI datasets, both generated using vRIC-seq. The SARS-CoV-2 dataset was derived from vRIC-seq data deposited in the Gene Expression Omnibus (GEO) under accession number GSE155733. The PDCoV dataset was obtained from GEO accession number GSE254992. The core principle of vRIC-seq is to capture nucleotide-resolution spatial interaction information within the native virion conformation via high-throughput sequencing, enabling systematic characterization of the intra-genomic RRI network and functional structural elements [1,16]. These two datasets were selected for three reasons. First, PDCoV and SARS-CoV-2 both belong to the Coronaviridae family but infect different host species, representing distinct evolutionary lineages within the coronavirus family. Second, both datasets were generated using the same vRIC-seq technology and processed with an identical computational pipeline, ensuring methodological consistency. Third, the two datasets are of comparable size and class balance, facilitating fair cross-method comparisons.

### 4.2. Data Processing and Sample Generation

vRIC-seq captures proximity-ligation contacts within mature virions [16]. Accordingly, the labels used in this study describe virion-associated RNA contact patterns and do not directly measure interaction dynamics in infected cells, tissue-specific environments, receptor engagement, or protease-dependent entry. We used the published vRIC-seq contact matrices and applied the same data-processing rule to both datasets. The matrices were inspected using Juicebox [17], and fragment pairs with an interaction score greater than 9 were retained according to the threshold used for the source data [18]. We did not perform additional RPM normalization, abundance-based downsampling, coverage-matched negative sampling, or an abundance-specific sensitivity analysis. Thus, residual associations with fragment abundance or local coverage cannot be excluded. Positive samples consisted of sequence fragments flanking retained contact sites. Negative pairs were randomly sampled from distinct genomic sites and excluded if they overlapped a positive pair [19]. Positive and negative samples were generated at a 1:1 ratio. The SARS-CoV-2 dataset comprised 57,101 pairs (28,552 positive and 28,549 negative), and the PDCoV dataset comprised 50,184 pairs (25,093 positive and 25,091 negative).

### 4.3. Sequence Encoding Strategies

This study employed three distinct sequence encoding strategies: one-hot encoding, which represents each nucleotide as a four-dimensional binary vector [20]; k-mer tokenization with Word2Vec embeddings, a distributed word representation trained on the Skip-gram architecture [21]; and a 1-mer-plus-motif tokenization scheme (NUCTokenizer) [22]. The choice of sequence encoding strategy directly influences the model’s ability to capture conserved RRI features and, consequently, determines cross-virus generalization performance. We therefore describe how each representation was generated before model training.

#### 4.3.1. One-Hot Sequence Encoding

For the one-hot encoding, each nucleotide was represented as a four-element binary vector [20]. The four canonical RNA bases (A, U, C, G) are encoded as follows:

(1) $$\left\{\begin{array}{c} A\to \left[1,0,0,0\right]\\ U\to \left[0,1,0,0\right]\\ G\to \left[0,0,1,0\right]\\ C\to \left[0,0,0,1\right] \end{array}\right.$$

In this study, one-hot encoding was applied to both traditional machine learning classifiers and deep learning architectures. For traditional classifiers (RF, SVM, KNN), each RNA sequence pair was independently one-hot encoded to generate two 4 × L matrices, which were then flattened into one-dimensional vectors and concatenated into a final feature vector of dimension 8 × L. This conversion made the sequence pair compatible with traditional classifiers, although it removed the original local arrangement of the sequence matrix. For deep learning models (CNN, RNN, and their variants), the one-hot encoding matrix was fed directly as an input tensor in its original 4 × L format. For CNN and RNN models, the matrix input was retained so that convolutional layers could scan local patterns and recurrent layers could process positions in sequence. The two encoding matrices from a sequence pair were typically merged through channel concatenation or early fusion into a single input.

#### 4.3.2. k-mer Tokenization and Word2Vec Embeddings

k-mer tokenization is a fundamental method for converting nucleotide sequences into discrete tokens. A k-mer is defined as a contiguous substring of length *k*. For an RNA sequence *S* of length *L*:

(2) $$S\;=\;\left(s_{1},\;s_{2},\;\ldots ,\;s_{L}\right)$$

The k-mer tokenization generates a token sequence:

(3) $$k\text{-}\mathrm{mers}\left(S\right)=\{s_{i}s_{i+1}\ldots s_{i+k-1}|1\leq i\leq L-k+1\}$$

k-mer tokenization can be classified as overlapping or non-overlapping depending on the stride. Overlapping k-mers retain all local patterns, whereas non-overlapping k-mers may discard boundary patterns [23]. In this study, Word2Vec and DNABERT used overlapping k-mer tokenization, while the NT used non-overlapping k-mer tokenization.

Word2Vec is a neural-network-based distributed word embedding method that maps discrete symbols into a continuous dense vector space, such that semantically similar symbols are positioned close together. We employed the Skip-gram architecture, which predicts the context words given a center word [21]. The objective function is:

(4) $$L=\frac{1}{T}\sum_{t=1}^{T}\sum_{-c\leq j\leq c,j\neq 0}\log P\left(w_{t+j}|w_{t}\right)$$

where *T* is the sequence length, *c* is the context window size, *w_t_* is the center word at position *t*, and *w_t+j_* is the context word at position *t + j*.

The Word2Vec approach treats k-mers as “words” and RNA sequences as “sentences” to learn distributed representations of k-mers. Each RNA sequence was decomposed into overlapping substrings of length k using a sliding window with a stride of one nucleotide, generating a vocabulary of all distinct k-mers appearing in the training corpus. A Word2Vec model was then trained using the Skip-gram architecture with a 50-dimensional embedding vector and a context window of 5. Sequence-level representation was obtained by averaging all k-mer embeddings within a given sequence. The primary advantage of Word2Vec embeddings lies in their ability to capture semantic similarity among k-mers, converting high-dimensional sparse k-mer frequency vectors into low-dimensional dense vectors [21,24]. However, static embeddings lack contextual awareness and cannot distinguish the same k-mer in different contexts, and the mean pooling operation discards the positional order information of k-mers within the sequence [25,26].

#### 4.3.3. NUCTokenizer (1-mer-Plus-Motif Tokenization)

NUCTokenizer is a custom nucleotide tokenizer with a vocabulary of 39 tokens, employing a motif-aware encoding strategy that assigns dedicated token identifiers to biologically relevant RNA sequence patterns [22]. The tokenizer covers three levels of sequence features: the first level comprises single nucleotides (1-mers), A, U, G, C, capturing individual base information; the second level comprises dinucleotide motifs (2-mers), including classical RNA base pairing patterns such as AU, GC, UG, and CG, capturing local base pairing information; the third level comprises short sequence patterns, including biologically relevant tetranucleotide motifs and other structural elements such as stem-loops, internal loops, and bulges associated with RNA secondary structure [22].

### 4.4. Model Architecture

#### 4.4.1. Traditional Machine Learning Baselines

To establish performance baselines and evaluate the effectiveness of different feature encoding schemes, we employed three classic machine learning algorithms as baseline classifiers, RF, SVM, and KNN, all using feature vectors generated by one-hot encoding or Word2Vec embeddings as input. Random forest was selected for its robustness against overfitting through ensemble voting across multiple decision trees, its ability to handle high-dimensional feature spaces, and its built-in feature importance estimation [27]. SVM was configured with a radial basis function (RBF) kernel, with default regularization parameter C and kernel coefficient gamma; the RBF kernel was chosen for its ability to model non-linear decision boundaries through implicit mapping to a high-dimensional feature space [28]. The KNN classifier was configured with the default number of neighbors k = 5 and uniform distance weighting, serving as a non-parametric, distance-based baseline method for evaluating the linear separability of feature representations generated by different encoding schemes [29].

#### 4.4.2. deepRAM Deep Learning Model

To investigate whether deep learning can overcome the limitations of traditional machine learning methods in RRI prediction, we adopted the deepRAM framework as a deep learning baseline. deepRAM is a modular deep learning architecture designed for nucleic acid sequence analysis, whose core design principle is decoupling the sequence encoding method from the neural network architecture, enabling fair evaluation of different encoding–architecture combinations through systematic pairing [30]. In this study, the framework provided six model configurations encompassing the full combination of two sequence encoding methods (one-hot encoding and Word2Vec k-mer embeddings) and three neural network architectures (CNN, RNN, and CNN_RNN). The CNN architecture employed a multi-scale convolutional design using kernels of different sizes to capture sequence motifs at multiple resolutions, followed by max-pooling layers for dimensionality reduction and a fully connected classification head. The RNN architecture utilized bidirectional long short-term memory (BiLSTM) networks to model sequence dependencies in both the 5′-to-3′ and 3′-to-5′ directions. The CNN_RNN architecture cascaded the convolutional feature extraction module with the recurrent sequence modeling module, combining the local pattern detection capability of CNNs with the sequence context modeling ability of LSTMs.

#### 4.4.3. Pre-Trained RNA Large Language Models

To explore the potential of pre-trained language models in RRI prediction, we fine-tuned and evaluated three pre-trained models with distinct pre-training strategies and model scales: DNABERT, RNAErnie, and the NT. During fine-tuning, all models had most pre-trained parameters frozen, with only the top classification head and a small number of adaptation parameters updated to preserve pre-trained knowledge while adapting to the downstream RRI prediction task.

DNABERT is a pre-trained bidirectional encoder model designed for nucleic acid sequence understanding [31], adapted from the Bidirectional Encoder Representations from Transformers (BERT) architecture [32]. Unlike natural language BERT, which uses words or subwords as basic units, DNABERT’s core innovation lies in introducing a k-mer tokenization strategy that treats every k consecutive nucleotides as a single token, preserving local sequence pattern information while reducing sequence length. DNABERT was pre-trained using masked language modeling, randomly masking 15% of k-mer tokens in the input and training the model to predict the masked tokens from their context, thereby learning contextual semantic representations of nucleotide sequences. DNABERT offers multiple k-mer variants (k ∈ {3,4,5,6}), among which k = 6 achieves the best performance [31]; we therefore adopted the DNABERT-6 variant pre-trained on human genome and regulatory element sequences. For the RRI prediction task, the input for each RNA–RNA pair was constructed by concatenating the two sequences with a special token. Let *a* and *b* represent the two RNA sequences, then the concatenated sequence *X* is:

(5) $$X=\left[a;\left[\mathrm{SEP}\right];b\right]$$

where [SEP] is the sequence separator token, and the semicolon indicates ordered sequence concatenation.

**Figure 7:**
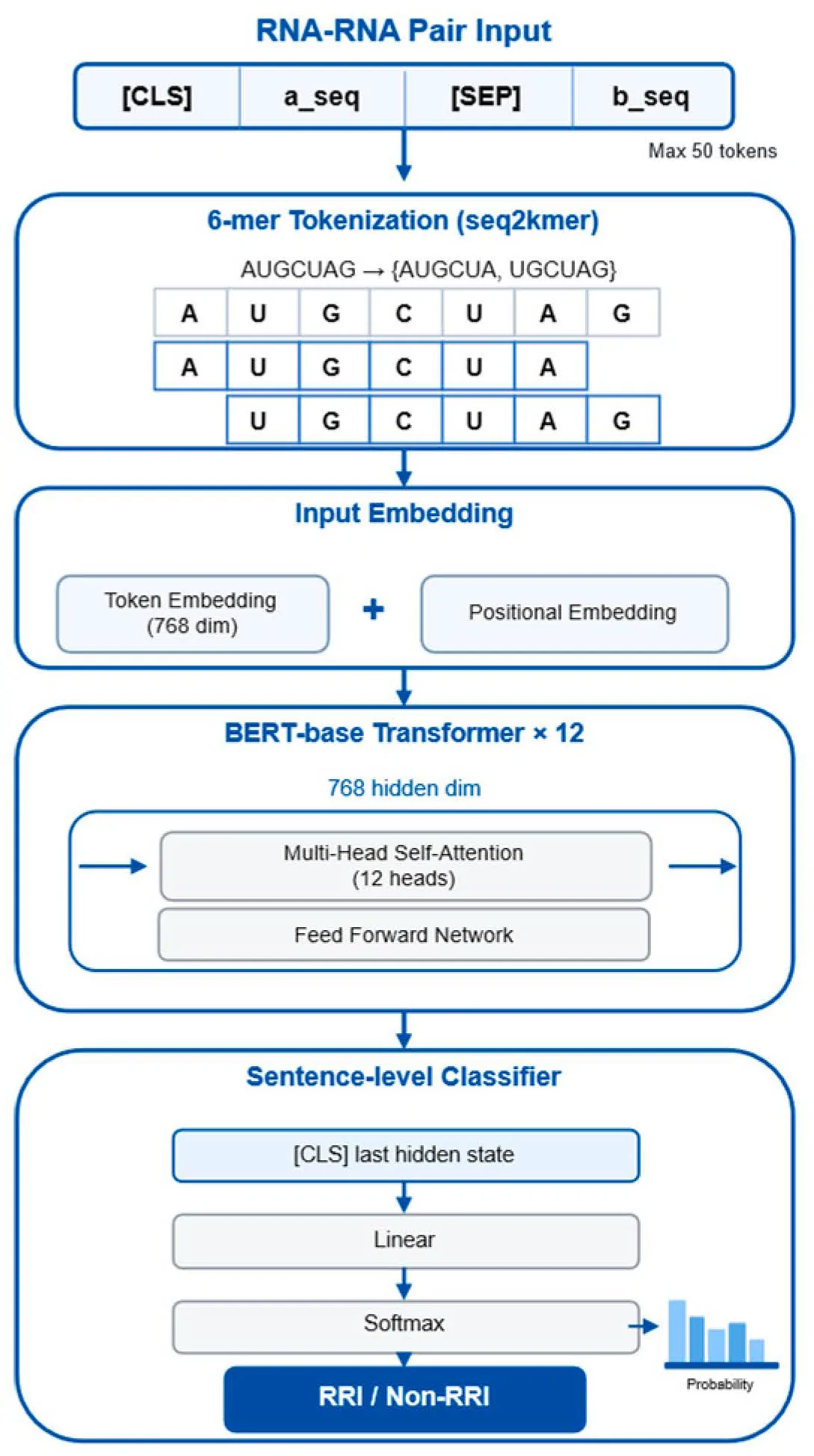
DNABERT model architecture for RRI prediction. RNA sequence pairs were input in the format [CLS] a_seq [SEP] b_seq with a maximum of 50 tokens. Input sequences were segmented using the seq2kmer method into overlapping 6-mers. Token embeddings and positional embeddings were added element-wise to form the DNABERT input representation, which was passed through a backbone network of 12 BERT-base Transformer encoder layers, each comprising multi-head self-attention and feed-forward neural networks with 768-dimensional hidden representations. The last-layer hidden state corresponding to the [CLS] token was extracted as the global representation of the sequence pair and passed through a linear layer and softmax classifier to predict RRI or Non-RRI.

The concatenated sequence was converted to overlapping 6-mers using the seq2kmer utility and then mapped to token IDs using the DNABERT tokenizer. Inputs were padded or truncated to a maximum length of 50 tokens. The classification head consisted of a single linear layer applied to the last-layer hidden representation (768-dimensional) with softmax activation, producing binary classification probabilities (Figure 7).

**Figure 8:**
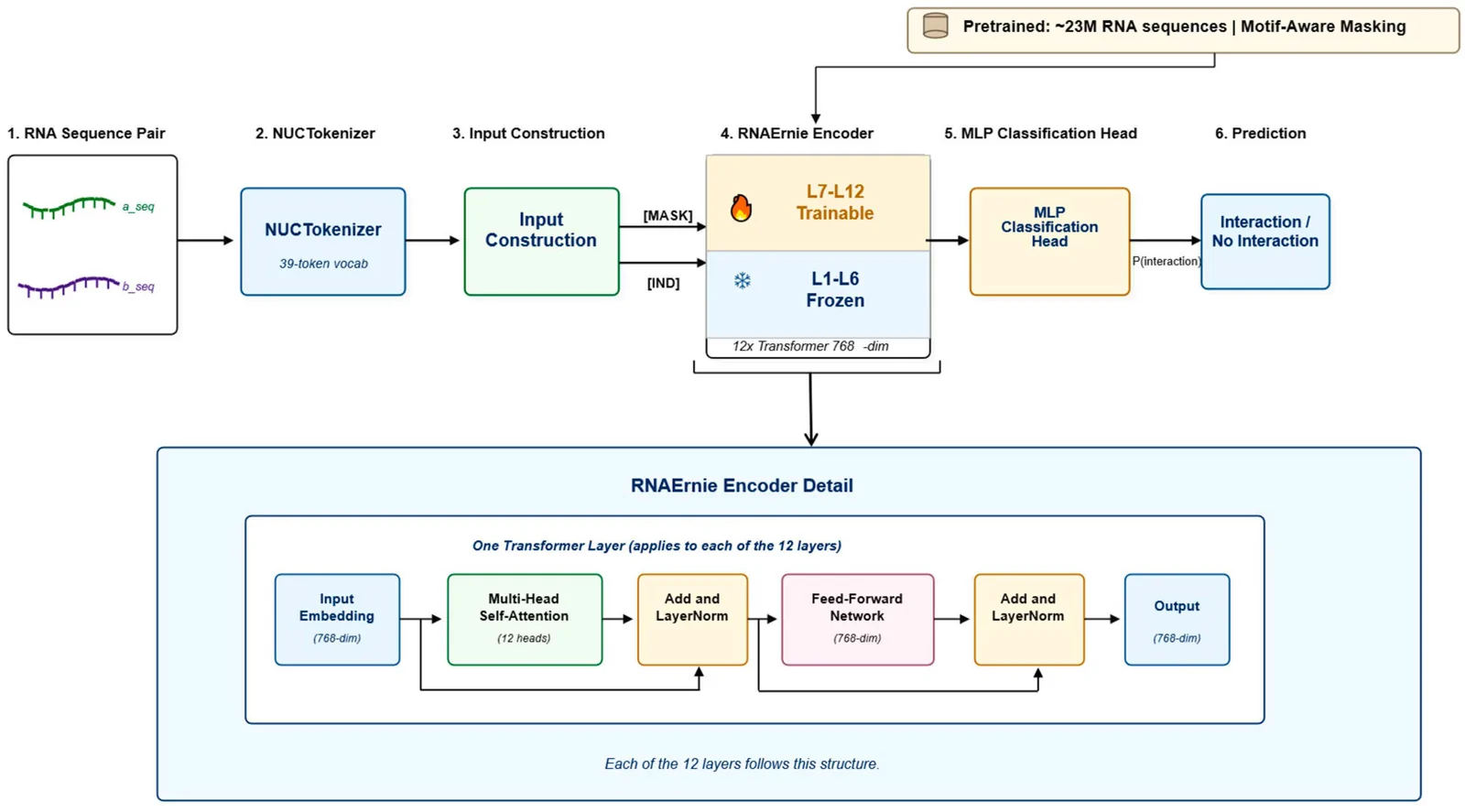
RNAErnie fine-tuning framework for coronavirus RRI prediction. The upper panel shows the main pipeline from input to prediction: paired RNA sequences (a_seq and b_seq) were first tokenized by NUCTokenizer using a 39-token vocabulary, with [MASK] and [IND] tokens introduced during input construction. The constructed input was then fed into the RNAErnie encoder, where the upper layers (L7–L12) participated in fine-tuning while the lower layers (L1–L6) remained frozen. The encoder output corresponding to the [IND] token served as the aggregated representation of the RNA sequence pair and was input to a multilayer perceptron (MLP) classification head to obtain binary predictions for RRI or non-interaction. The magnified region at the bottom illustrates the internal structure of the RNAErnie encoder, including the frozen/trainable partition of 12 Transformer layers, and the core components of a single Transformer layer: input embedding, multi-head self-attention, residual normalization, feed-forward network, and output representation.

RNAErnie is an RNA sequence pre-trained language model built on the Enhanced Representation through kNowledge IntEgration (ERNIE) framework [22,33]. Its encoder comprises 12 Transformer layers, 12 attention heads, and 768-dimensional hidden representations, totaling approximately 95 million parameters, comparable to DNABERT in parameter scale but fundamentally different in architectural design (Figure 8). The key distinction of RNAErnie lies in its multi-level pre-training strategy. Unlike standard BERT, which employs only random token-level masking, the ERNIE framework introduces a knowledge-enhanced masking mechanism that enables the model to capture both sequence-level syntactic structure and motif-level biological semantics during pre-training. RNAErnie uses NUCTokenizer for input tokenization, which has a vocabulary of 39 unique tokens employing the 1-mer-plus-motif tokenization approach. For the RRI prediction task, the input representation was constructed by inserting a [MASK] token between the two RNA sequences to serve as an interaction signal, with its final hidden representation used to predict whether the two sequences interact. The classification head appends a special [IND] token to the input sequence, utilizing its corresponding hidden representation for binary classification. The model employed a two-stage fine-tuning protocol: the first stage performed end-to-end fine-tuning with a coarse-grained binary classification objective; the second stage adopted a top-k ensemble classification strategy, selecting the most confident top-k prediction samples from the first stage to refine the decision boundary and improve classification robustness.

**Figure 9:**
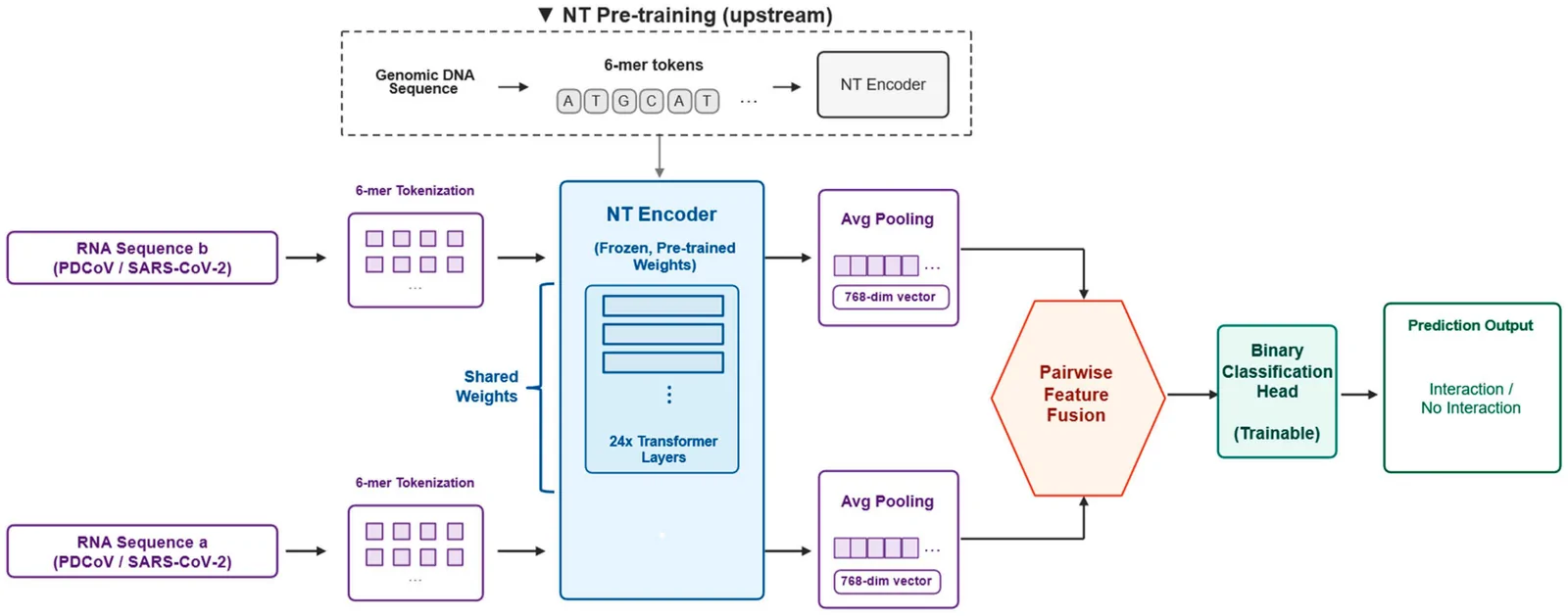
Coronavirus RRI prediction framework based on the pre-trained NT. Input RNA sequences a and b (PDCoV or SARS-CoV-2) were first subjected to 6-mer tokenization, and sequence representations were extracted through a shared-weight, frozen NT encoder. Average pooling was applied to obtain 768-dimensional vector representations, followed by a four-way feature fusion module to integrate the dual-sequence features. The fused representation was passed through a trainable binary classification head to determine whether the two RNA sequences interact and output the interaction probability.

NT is a model pre-trained on large-scale genome sequences [34]. We used the NT-v2 250M Multi-Species variant, whose Transformer encoder comprises 24 layers, 16 attention heads, and 768-dimensional hidden representations. NT was pre-trained using a masked language modeling objective on a large-scale corpus of human and multi-species genome sequences, with a pre-training data scale substantially exceeding that of DNABERT and RNAErnie. During pre-training, the model learned to predict masked 6-mer tokens, establishing a general understanding of nucleotide sequence patterns across large-scale genomic data [34]. For the RRI prediction task, because the original NT architecture only supports single-sequence input, we designed a custom four-way feature fusion classification head to accommodate dual-sequence input (Figure 9). The two input RNA sequences were independently encoded by the NT encoder, each generating a 768-dimensional sequence representation. These representations were deeply fused through four complementary computations: concatenation (preserving original information), element-wise absolute difference (quantifying sequence divergence), element-wise product (characterizing interaction relationships), and element-wise summation (extracting additive responses). The outputs of these four operations were concatenated into a 3072-dimensional fused feature vector, which was then fed into a multi-layer perceptron (MLP) with rectified linear unit (ReLU) activation, followed by a sigmoid layer for binary classification.

### 4.5. Evaluation Framework

A comprehensive evaluation framework was designed to assess model performance from two complementary perspectives: within-dataset discriminative ability and cross-dataset generalization capability. For within-dataset evaluation, five-fold stratified cross-validation [35] was independently performed on each dataset with a fixed random seed. For cross-dataset generalization evaluation, the model was trained on the complete training set of one virus and evaluated on the complete dataset of the other virus, with no cross-contamination between samples. Unlike within-dataset evaluation, cross-dataset experiments did not employ cross-validation; instead, the entire source dataset was used for training and the entire target dataset for evaluation, providing models with the maximum amount of training data. Within-dataset evaluation on PDCoV was performed for traditional machine learning classifiers and deepRAM deep learning models, while pre-trained language models underwent SARS-CoV-2 within-dataset evaluation, PDCoV within-dataset evaluation, and cross-dataset generalization evaluation.

We employed area under the receiver operating characteristic curve (AUC), accuracy, precision, recall, and F1-score as evaluation metrics to comprehensively characterize binary classification performance from three dimensions: overall discriminative ability, classification balance, and threshold sensitivity. Accuracy, precision, recall, and F1-score are defined as follows:

(6) $$\mathrm{Accuracy}=\frac{TP+TN}{TP+TN+FP+FN}$$

(7) $$\mathrm{Precision}=\frac{TP}{TP+FP}$$

(8) $$\mathrm{Recall}=\frac{TP}{TP+FN}$$

(9) $$F1=\frac{2\times \mathrm{Precision}\times \mathrm{Recall}}{\mathrm{Precision}+\mathrm{Recall}}$$

where *TP*, *TN*, *FP*, and *FN* denote true positives, true negatives, false positives, and false negatives, respectively.

Since the positive-to-negative sample ratios in both datasets are approximately 1:1, accuracy reliably reflects overall classification ability. Meanwhile, threshold-independent AUC was used as the core benchmark to ensure fairness of model comparisons. Given the inherent trade-off between precision and recall, we further included F1-score as a comprehensive measure to avoid evaluation bias from any single metric.

## Figures and Tables

**Table 1:** Prediction results of traditional machine learning models on the PDCoV dataset.

Encoding	k-mer	Classifier	Precision	Recall	F1-Score	Accuracy	AUC
OneHot	-	RF	0.7489	0.8047	0.7758	0.7674	0.8543
OneHot	-	SVM	0.7352	0.8211	0.7758	0.7627	0.8401
OneHot	-	KNN	0.6809	0.8222	0.7449	0.7185	0.8070
Word2Vec	3	RF	0.8797	0.6379	0.7396	0.7753	0.8600
Word2Vec	3	SVM	0.8492	0.6962	0.7651	0.7863	0.8648
Word2Vec	3	KNN	0.7573	0.8809	0.8144	0.7993	0.8995
Word2Vec	4	RF	0.8891	0.6076	0.7219	0.7659	0.8574
Word2Vec	4	SVM	0.8535	0.7105	0.7755	0.7943	0.8736
Word2Vec	4	KNN	0.7561	0.8841	0.8151	0.7994	0.9017
Word2Vec	5	RF	**0.9070**	0.5959	0.7193	0.7674	0.8635
Word2Vec	5	SVM	0.8608	0.6964	0.7700	0.7919	0.8723
Word2Vec	5	KNN	0.7553	**0.8901**	**0.8172**	**0.8009**	**0.9055**
Word2Vec	6	RF	0.8990	0.5700	0.6977	0.7530	0.8268
Word2Vec	6	SVM	0.8527	0.6518	0.7389	0.7696	0.8365
Word2Vec	6	KNN	0.7258	0.8839	0.7971	0.7750	0.8912

The metrics reported in the table are the mean values obtained from five-fold cross-validation. The Word2Vec k-mer results include k = 3–6. Among the traditional machine learning methods, the highest AUC was achieved by the KNN classifier using k = 5 Word2Vec representations. Boldface indicates the highest value in each column.

**Table 2:** deepRAM model prediction results on the PDCoV dataset.

Encoding	Architecture	Precision	Recall	F1-Score	Accuracy	AUC
OneHot	CNN	0.8118	0.7522	0.7805	0.7886	0.8702
OneHot	CNN_RNN	0.7410	0.7311	0.7354	0.7374	0.8118
OneHot	RNN	0.8292	0.8375	0.8332	0.8323	0.9049
Word2Vec	CNN	**0.8728**	0.8476	**0.8600**	**0.8620**	**0.9318**
Word2Vec	CNN_RNN	0.8475	**0.8527**	0.8499	0.8495	0.9209
Word2Vec	RNN	0.8481	0.8496	0.8488	0.8486	0.9214

The table presents the Precision, Recall, F1-score, Accuracy, and AUC obtained by combining OneHot and Word2Vec representations with CNN, CNN_RNN, and RNN architectures. Boldface indicates the best-performing value for each metric.

**Table 3:** Within-dataset prediction results of pre-trained language models on PDCoV and SARS-CoV-2 datasets.

Dataset	Model	Precision	Recall	F1-Score	Accuracy	AUC
PDCoV	DNABERT	0.8933	0.8928	0.8928	0.8928	0.9595
PDCoV	RNAErnie	0.8271	0.8263	0.8262	0.8263	0.9176
PDCoV	NT	0.6614	0.6739	0.6671	0.6640	0.7182
SARS-CoV-2	DNABERT	0.9188	0.9187	0.9187	0.9187	0.9741
SARS-CoV-2	RNAErnie	0.8775	0.8770	0.8770	0.8770	0.9490
SARS-CoV-2	NT	0.6965	0.7376	0.7158	0.7074	0.7734

The table presents the Precision, Recall, F1-score, Accuracy, and AUC of DNABERT, RNAErnie, and NT.

**Table 4:** Cross-dataset prediction results of pre-trained language models.

Model	Training	Testing	Precision	Recall	F1-Score	Accuracy	AUC	AUC Retention
DNABERT	PDCoV	SARS-CoV-2	**0.7944**	**0.7862**	**0.7847**	**0.7862**	**0.8372**	0.8595
DNABERT	SARS-CoV-2	PDCoV	0.7972	0.7675	0.7617	0.7675	0.8116	0.8459
RNAErnie	PDCoV	SARS-CoV-2	0.7720	0.7682	0.7673	0.7682	0.8171	0.8610
RNAErnie	SARS-CoV-2	PDCoV	0.7791	0.7581	0.7535	0.7581	0.7994	**0.8712**
NT	PDCoV	SARS-CoV-2	0.5313	0.4049	0.4590	0.5237	0.5314	0.6871
NT	SARS-CoV-2	PDCoV	0.5486	0.4054	0.4654	0.5356	0.5494	0.7650

Training dataset, testing dataset, precision, recall, F1-score, accuracy, AUC, and AUC retention ratio are listed. AUC retention is defined as cross-dataset AUC divided by the target dataset within-dataset AUC. Boldface indicates the highest value in each column.

## Data Availability

The datasets analyzed in this study are publicly available in the Gene Expression Omnibus (GEO) under accession numbers GSE155733 and GSE254992. Additional data supporting the findings of this study are available from the corresponding author upon reasonable request. Source code and data have been deposited in Zenodo and are available via the following link: https://doi.org/10.5281/zenodo.22025445 (accessed on 17 August 2026).
